# Crosstalk Suppression in a Multi-Channel, Multi-Speaker System Using Acoustic Vector Sensors

**DOI:** 10.3390/s25216731

**Published:** 2025-11-03

**Authors:** Grzegorz Szwoch

**Affiliations:** Department of Multimedia Systems, Faculty of Electronics, Telecommunication and Informatics, Gdańsk University of Technology, 80-233 Gdańsk, Poland; grzszwoc@pg.edu.pl

**Keywords:** crosstalk, acoustic vector sensor, diarization, speech recognition

## Abstract

Automatic speech recognition in a scenario with multiple speakers in a reverberant space, such as a small courtroom, often requires multiple sensors. This leads to a problem of crosstalk that must be removed before the speech-to-text transcription is performed. This paper presents an algorithm intended for application in multi-speaker scenarios requiring speech-to-text transcription, such as court sessions or conferences. The proposed method uses Acoustic Vector Sensors to acquire audio streams. Speaker detection is performed using statistical analysis of the direction of arrival. This information is then used to perform source separation. Next, speakers’ activity in each channel is analyzed, and signal fragments containing direct speech and crosstalk are identified. Crosstalk is then suppressed using a dynamic gain processor, and the resulting audio streams may be passed to a speech recognition system. The algorithm was evaluated using a custom set of speech recordings. An increase in SI-SDR (Scale-Invariant Signal-to-Distortion Ratio) over the unprocessed signal was achieved: 7.54 dB and 19.53 dB for the algorithm with and without the source separation stage, respectively.

## 1. Introduction

Automatic Speech Recognition (ASR) systems transcribe spoken words into written text. An ASR system is an invaluable tool for creating speech transcripts in practical scenarios such as courtroom sessions, business meetings, physician-patient interviews, etc. In the past, ASR pipelines were based on feature extraction and statistical models, such as Hidden Markov Models and Gaussian Mixture Models, with limited efficiency [1,2]. With the advent of machine learning methods, ASR systems started developing rapidly. Modern ASR solutions are typically powered by deep learning architectures, combining acoustic modeling, language modeling, and increasingly large-scale self-supervised pretraining [3,4,5]. Current AI-based ASR systems often work on the end-to-end principle, processing raw audio streams and outputting the recognized text, assigned to specific speakers. A typical ASR processing pipeline consists of stages such as signal pre-processing, speaker detection and diarization (who spoke when), separation (extraction of individual speakers), and transcription (speech-to-text conversion).

Most of the modern ASR systems based on artificial intelligence (AI) operate on single-channel audio streams. However, in a practical scenario such as a courtroom session, multiple speakers are present in a reverberant room, at some distance between each other. Usually, it is not possible to achieve a sufficient signal-to-noise ratio (SNR) for an accurate speech transcription with an ASR system using a single microphone. Therefore, multiple sensors are often placed across the room, close to each speaker. This creates another problem: each sensor records not only direct speech from the near speaker, but also crosstalk from all the other speakers. If each audio channel is processed by the ASR independently, without pre-processing, then not only will the transcribed speech be duplicated across the channels, but also signal attenuation and distortion (from reverberation and the background noise) present in the crosstalk segments may lead to speech recognition errors, and they increase the risk of AI model hallucinations (producing wrong or non-existing text). Therefore, it is viable to pre-process the raw audio streams to remove crosstalk from the signals and to provide the ASR system with signals containing only direct speech.

The problem of crosstalk reduction in a multi-speaker system was initially approached with statistical methods, such as Hidden Markov Models [6] and Gaussian Mixture Models [7]. In more recent publications, Meyer et al. used frequency domain adaptive filtering [8] and a Kalman-based Wiener filter approach [9] for multi-channel crosstalk reduction. Later developments of deep neural networks (DNN) shifted the focus to using AI models for crosstalk reduction. Milner and Tain [10] trained DNNs on features derived from the concatenation of speaker channel features to detect which channel is correct for each frame. Sell and McCree [11] investigated I-vector training and extraction with the presence of crosstalk and found that crosstalk detection and multi-speaker detection may be different tasks, requiring separately trained detectors. Lin et al. [12] developed a multi-channel directional ASR model that processes multiple beamformer outputs simultaneously and combines that model with serialized output training, using signals recorded by a microphone array in smart glasses. Wang et al. [13] proposed linear filtering of the unsupervised or weakly supervised DNN estimates to cancel out crosstalk. Han et al. [14] created a crosstalk rejection framework based on a multi-channel voice activity detector and a model trained on synthetic recordings.

In recent AI-based end-to-end ASR systems, the processing pipeline combines stages such as crosstalk reduction, diarization, and speaker separation [15,16]. Multi-channel diarization is especially important, as it can identify speech segments related to direct speech and crosstalk. Traditional diarization methods were based on speech processing algorithms, such as Gaussian Mixture Models [17,18]. More recent publications focus on employing deep learning algorithms [19,20]. Chang et al. [21] used Transformer architecture and external dereverberation preprocessing in a multi-speaker ASR system. Medennikov et al. [22] created a target-speaker voice activity detection system for multi-speaker diarization in a dinner party scenario. Raj et al. [23] proposed an end-to-end modular system that combines independently trained separation, diarization, and recognition components. Zheng et al. [24] developed two multi-channel diarization systems, with and without direction of arrival estimation, which have enhanced capability in detecting overlapped speech and identifying speakers via learning spatial features. Horiguchi et al. [25] enhanced an end-to-end neural diarization system by using multi-channel signals from distributed microphones. Gomez et al. [26] performed speaker diarization and identification from a single-channel recording by creating a virtual array of microphones. Wang et al. [27] described a spatial-aware speaker diarization system for the multi-channel multi-party meeting, which obtains direction information of the speaker by microphone array using speaker spatial embedding derived from superdirective beamforming. Taherian and Wang [28] introduced an end-to-end speaker separation via a neural diarization system to identify the speech activity of each speaker. Wang et al. [29] proposed a three-stage modular system to enhance a single-channel neural speaker diarization and recognition system by utilizing spatial cues from multi-channel speech. Ma et al. [30] presented a spatial long-term iterative mask estimation method utilizing spatial information to improve the speaker diarization performance in various real-world acoustic scenarios. Xylogiannis et al. [31] evaluated a framework combining speaker embeddings with Time Difference of Arrival (TDOA) values from available microphone sensor arrays in meetings and concluded that integration of spatial information can significantly improve the performance of state-of-the-art deep learning diarization models. Cord-Landwehr et al. [32] proposed a spatio-spectral, combined model-based and data-driven diarization pipeline consisting of TDOA-based segmentation followed by embedding-based clustering capable of correctly tracking speakers when they change positions.

Most ASR systems and diarization methods operate on audio signals recorded with standard microphones. There are also published works that process signals obtained from multi-microphone setups. Acoustic Vector Sensors (AVS) can provide information on the direction of arrival (DOA) of the sound waves [33]. Therefore, they can be useful in the ASR algorithms in which speaker detection and diarization are based on spatial data. Shujau et al. [34] demonstrated how the directional characteristics of AVS can be used to separate speech sources. Jin et al. [35] performed robust speaker DOA estimation based on the inter-sensor data ratio model and binary mask estimation in the bispectrum domain, under directional non-speech interference and non-directional background noise, using AVS. Zou et al. [36] investigated the multichannel signal properties of a single AVS to obtain the inter-sensor data ratio model in the time-frequency domain, estimate DOA, and enhance the target speech. Wang et al. [37,38] used AVS in a multi-speaker DOA estimation system based on DNN. Geng et al. [39] introduced a pipeline for selecting reliable time-frequency bins and estimating multi-source DOAs using a single AVS. Gburrek et al. [40] proposed a diarization system that extracts TDOA from spatial information obtained with an acoustic sensor network to estimate speakers’ activity.

The problem of crosstalk in a multi-channel ASR system may be approached in two ways. It is possible to train an end-to-end AI model to handle multiple input audio streams with crosstalk, producing an accurate transcript. This approach is under investigation by various researchers, but the complexity of such a system and the need for a vast training dataset for this task lead to the fact that, currently, there is no working system of this kind ready to be used in practical applications. As an alternative, a method of pre-processing of the input audio streams that leads to crosstalk suppression may be proposed. After the pre-processing, audio streams without crosstalk may be processed independently by the current ASR systems, and the speech recognition results may be combined to obtain a complete transcript. The algorithm presented in this paper utilizes the second approach.

The proposed method of crosstalk reduction in a multi-channel system uses signals recorded with several AVSs placed in a room, near the speakers. In the previous publication [41], it was shown that time-frequency processing of a single AVS signal allows for an efficient separation of two speakers, and that the resulting signals are suitable for the ASR. In this paper, signals from multiple sensors are processed to detect near speakers and their directions, and then to detect and suppress crosstalk, leaving only direct speech for the ASR system. The contribution of this paper is a method of joint analysis of signals from multiple AVSs with multiple active speakers, an algorithm for detecting speakers’ orientation relative to the sensors, and for detecting crosstalk in the signal and suppressing it. The proposed algorithm is based on the ability of an AVS to detect the speaker’s DOA and to determine signal components originating from directions not related to direct speech.

The rest of the paper is organized as follows. Section 2 describes the proposed algorithm: an overview, sound intensity calculation, speaker detection, source separation, crosstalk detection, and suppression. Section 3 presents the dataset created for the evaluation, the evaluation metric SI-SDR, the reference system, and the experimental results. Additionally, a case of overlapping speech is presented. The obtained results and the performance of the proposed algorithm are discussed in Section 4, and the paper ends with Section 5.

## 2. Materials and Methods

The algorithm for crosstalk suppression in a multi-speaker system with multiple AVS will be presented in detail, starting with a description of the problem to solve and the general idea of the algorithm, and proceeding to a detailed description of each processing stage.

### 2.1. An Overview of the Crosstalk Suppression Algorithm

The problem is defined as follows. The goal is to obtain a speech transcript in a scenario with multiple speakers positioned across a reverberant space (such as in a courtroom session). To achieve this result, several AVSs are placed within a room, close to the speakers. The number of active speakers and their positions relative to the sensors is not known a priori. All the sensors record synchronized audio streams. Each recorded audio stream (a channel) contains direct speech (if the near speaker is active), crosstalk (when far speakers are active), and background noise. The task of the algorithm is to detect and suppress crosstalk in all channels. Specifically, the algorithm must: (1) detect the number of active speakers in the recording and their direction relative to the sensors, (2) detect the signal fragments representing near speech and crosstalk in every channel, and (3) suppress crosstalk and possibly also background noise from all channels, leaving only direct speech. The processing must be performed such that the output streams are suitable for ASR, allowing for sufficient speech recognition accuracy.

In order to focus on a specific problem, it was decided that the formal evaluation of the algorithm described in this paper (i.e., calculation of the performance metrics) would be performed in a scenario in which speakers are active in turns, i.e., there are no speech segments with multiple speakers active (overlapping speech). Speaker separation in overlapping speech is a separate and complex problem that requires a different testing methodology, and it is outside the scope of this paper. However, the AVS algorithm is also able to perform speaker separation in overlapping speech, as presented in the previous publication [41], and a brief demonstration (without formal testing) will be presented further in this paper.

**Figure 1:**
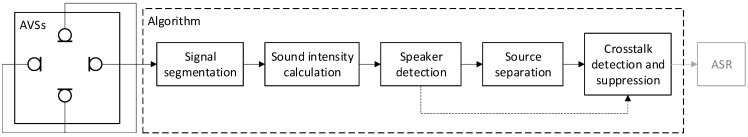
Block diagram of the multi-channel crosstalk suppression algorithm.

An overview of the proposed algorithm is presented in Figure 1. The consecutive processing stages, starting with acquiring input streams from the sensors and finishing with passing the processed (crosstalk-free) streams to an ASR system, will be presented in the following subsections. The actual speech recognition is not a part of the proposed algorithm; it is realized by an external ASR system.

### 2.2. Acoustic Vector Sensor

An Acoustic Vector Sensor (AVS) is a device that measures both the pressure and the particle velocity components of an acoustic field. Unlike conventional microphones, which capture only scalar sound pressure, AVS provides vector information by detecting the direction of sound propagation. This is achieved using a setup of transducers: a pressure sensor (microphone) and two or three orthogonally oriented particle velocity sensors. By capturing both magnitude and directional information, AVSs allow for sound source localization and noise suppression. In the scenario described in this paper, multiple AVSs are used to capture the sound field across the room. Particle velocity is measured in the horizontal plane, in X-Y directions. Since true particle velocity sensors are expensive, this quantity is often approximated with a pressure gradient, computed from the pressure signals recorded by two identical, closely spaced, omnidirectional microphones. For the *X* axis, pressure *p_X_*_1_, *p_X_*_2_ is measured with two microphones placed on this axis, and converted to the sensor pressure *p_X_* and particle velocity *u_X_* as follows:

(1) $$p_{X}\left(t\right)=\left(p_{X1}\left(t\right)-p_{X2}\left(t\right)\right)/2,$$

(2) $$u_{X}\left(t\right)=p_{X1}\left(t\right)-p_{X2}\left(t\right).$$

Similar operations are performed for the *Y* axis. Pressure signals *p_X_*, *p_Y_* calculated for two axes are averaged into a single signal *p*. Therefore, each AVS provides a three-channel signal: (*p*, *u_X_*, *u_Y_*).

Further processing is performed in the digital domain. Streams of signal samples are partitioned into overlapping blocks, and each block is transformed into the frequency domain using the Discrete Fourier Transform. From the frequency (spectral) representations *P*(*ω*), *U_X_*(*ω*) of the pressure and particle velocity signals, sound intensity *I_X_*(*ω*) in the *X* axis can be computed [42]:

(3) $$I_{X}\left(\omega \right)=Re\left\{P(\omega )\cdot U_{X}^{*}(\omega )\right\},$$

where the asterisk denotes complex conjugation. Sound intensity in the *Y* axis is computed accordingly.

Sound intensity is a vector quantity describing the energy flow in sound waves along a given axis. Total sound intensity *I*(*ω*) is a scalar quantity expressing the amount of acoustic energy measured with the AVS, regardless of the direction, and is calculated as follows:

(4) $$I\left(\omega \right)=\sqrt{I_{X}^{2}\left(\omega \right)+I_{Y}^{2}\left(\omega \right)}.$$

### 2.3. Speaker Detection

The first stage of the algorithm detects the number of active speakers and their orientation relative to the sensors. Given the intensity signals *I_X_*(*ω*) and *I_Y_*(*ω*) computed in the frequency domain for a block of signal samples, the horizontal direction (the azimuth) *φ*(*ω*) of the sound source can be obtained:

(5) $$\varphi \left(\omega \right)=arctan\frac{I_{X}(\omega )}{I_{Y}(\omega )}.$$

A recording from a single AVS is partitioned into the overlapping segments, and the azimuth is computed for each segment separately. Statistical analysis of the azimuth from all the signal blocks is then performed to determine the directions of dominant sound sources. The azimuth range 0° to 360° is divided into subranges (bins). In the experiments described here, bins of 5° width were used (72 bins in total). For each bin defined by the edge azimuth values (*φ*_min_, *φ*_max_), the total sound intensity of all frequency components having the azimuth within the bin range is calculated:

(6) $$I_{\left(\varphi min,\varphi max\right)}=\frac{1}{N}\sum_{n=1}^{N}\sum_{k=kmin}^{kmax}I_{n,k}\left(\omega \right)\;\left|\;\varphi min\leq \varphi_{n,k}\left(\omega \right)<\varphi max\right.,$$

where *N* is the number of the analyzed blocks, *k*min and *k*max are the indices of the first and the last spectral bin used in the analysis.

The procedure described above effectively computes a histogram of the azimuth weighted by the total intensity. The values *k*min and *k*max define the frequency range used for the detection of sound source direction. In the experiments described here, frequency range from 93.75 Hz to 3984 Hz (spectral bins 4 to 170 for a signal sampled at 48 kHz with a block size of 2048 samples) was analyzed. Frequencies beyond that range were excluded from the analysis because of low SNR: for the low frequencies, noise is predominant in the signal; for the high frequencies, the energy of a speech signal is too low.

Initially, this approach was applied to each channel individually. However, since all active sound sources may be detected in every channel, it was not possible to discern the near speaker from crosstalk, which led to incorrect results. Since the signals from all sensors are recorded simultaneously, the algorithm was extended to detect the dominant channel. In each signal block, for each AVS, the channel intensity *I_ch_* is calculated:

(7) $$I_{ch}[n]=\alpha_{I}\;\cdot I_{ch}\left[n-1\right]+\left(1-\alpha_{I}\right)\cdot \sum_{k=kmin}^{kmax}I_{n,k}\left(\omega \right),$$

where *n* is the index of the signal block, *α_I_* is the exponential smoothing factor (0.85 in the described experiments).

In order to detect only the dominant sound sources (near speakers), channel intensity is calculated for each signal block and for each sensor, and the channel with the highest *I_ch_* value is selected as the dominant one in each block. Only the histogram of the dominant channel in the current block is updated. It is assumed that the direct signal from the near speaker is present in the dominant channel. Therefore, the analysis only detects the near speakers in each channel, and not crosstalk. With this approach, it is also possible to exclude channels without near-speaker activity from further processing.

In the next stage, directions of sound sources are found from the histograms. The histogram values are smoothed by circular convolution with a Gaussian filter (kernel size equal to 5 in the presented case). Each histogram is expected to contain local maxima (modes), one for each detected sound source. The modes are extracted from the histogram by finding azimuth ranges around the local maxima that are above the threshold, equal to the mean of the histogram multiplied by a chosen factor (a tunable parameter, e.g., 2). Each mode is described by the azimuth and the intensity of its maximum, and the azimuth range of histogram values above the threshold.

**Figure 2:**
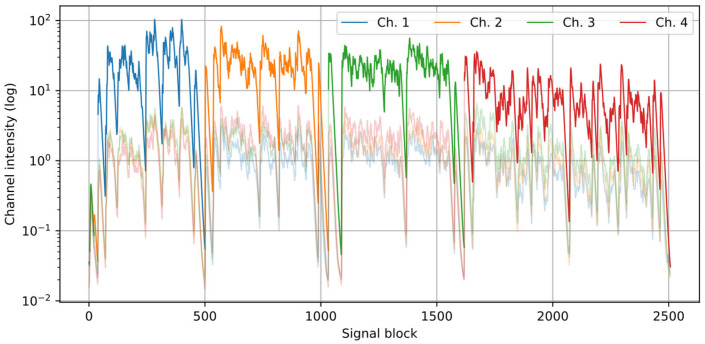
Channel intensity *I_ch_* plots for an example recording in which four speakers were active in turns. Dark colors indicate the signal fragments during which the channel was dominant, light colors—the remaining signal parts.

**Figure 3:**
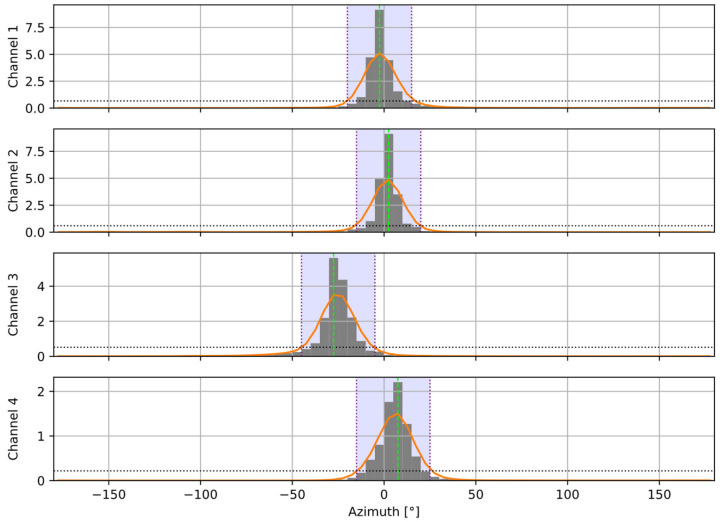
Azimuth histograms calculated for the same recording as in Figure 2. Gray bars show raw histogram values, orange lines—smoothed histograms. Horizontal dotted lines are the detection thresholds. Vertical dotted lines and the violet background indicate the detected modes (azimuth ranges) for the individual speakers.

Figure 2 and Figure 3 present an example of the results from the speaker detection algorithm, using one of the recordings from the dataset described further in the paper. A setup of four channels was used, and four speakers (one per channel) were active in turn. Figure 2 shows that the proposed procedure correctly establishes the active speaker in the dominant channel. Figure 3 presents the calculated histograms of the azimuth and the modes (one per a near speaker) extracted from the histograms.

In the presented examples, one speaker was present in each channel. The proposed method can detect multiple speakers per channel, as long as a sufficient angular distance between the sources is maintained (in practice: at least 30°).

### 2.4. Source Separation

Using the spatial data obtained during the previous processing stage, near speakers can be separated from crosstalk and noise sources, using a method of time-frequency bin selection [34]. The algorithm for source separation using a single AVS was presented in detail in the previous publication [41]. Here, each channel is processed separately. The azimuth range (*φ_low_*, *φ_high_*) is found for each speaker using the procedure described earlier (the azimuth values must be unwrapped, because they wrap at 360°). For each signal block, azimuth *φ*(*ω*) is calculated in the frequency domain (Equation (5)), and a binary mask *M*(*ω*) is computed:

(8) $$M\left(\omega \right)=\left\{\begin{array}{l} 1,\;if\;\varphi_{low}\leq \varphi \left(\omega \right)<\varphi_{high}\\ 0,\;otherwise \end{array},\right.$$

then the spectrum of the pressure signal recorded with the AVS is processed:

(9) $$P^{\prime }\left(\omega \right)=M\left(\omega \right)\cdot P\left(\omega \right).$$

This operation effectively zeroes frequency bins having the azimuth beyond the detected range (*φ_low_*, *φ_high_*) for a selected source.

The results of processing all overlapping blocks of the signal are then transformed back to the time domain, and the signal is reconstructed using the overlap-add method. The result of processing is a single-channel pressure signal, with suppressed energy from unwanted sound sources, such as crosstalk and noise.

In the example presented in Figure 2 and Figure 3, the speakers are active in turn, so the separation procedure is not strictly needed in the presented processing pipeline. However, in the case of overlapping speech (multiple speakers active) or in the presence of strong noise sources, the separation procedure is necessary to filter out the unwanted sound sources from each channel.

### 2.5. Crosstalk Detection and Suppression

The results of signal analysis performed within the previous stages of the algorithm can now be used to detect signal segments containing crosstalk and to suppress it. Even if the source separation procedure described earlier is applied, the degree of crosstalk suppression is usually not sufficient. Due to the presence of reverberation in a room, some reflected sound waves from far speakers may be acquired by the sensor from the same direction as the near speaker. Therefore, residual crosstalk may remain in the signal, and its level depends on the acoustic properties of the room, loudness of speech, etc. Since the ASR models are usually very sensitive in audio signal detection, they tend to detect even low-level audio fragments, and because the residual crosstalk is distorted, this may result in AI model hallucinations (generating incorrect or non-existing text). Therefore, an additional stage of crosstalk detection and suppression is needed.

Pressure signals recorded with the AVSs are processed independently for each channel, in overlapping blocks, the same way as during the previous stages. To determine whether a signal block contains mostly near speech or crosstalk, a coverage metric *c* is calculated from the binary mask calculated during the previous stage:

(10) $$c=\frac{\sum_{k=kmin}^{kmax}M\left(\omega \right)\;}{kmax-kmin},$$

The coverage metric *c* is a value normalized to the (0, 1) range, and it is expected to be high when direct speech is dominant in the signal, and low if mostly crosstalk and noise are present. Therefore, crosstalk may be detected by thresholding the coverage metric.

The proposed algorithm realizes a ‘crosstalk gate’, utilizing a similar concept to a noise gate—a dynamic processing algorithm that suppresses noise in the audio signal [43]. The gate has a gain factor *g* = 1 if direct speech was detected (the gate is open), and *g* = 0 otherwise (the gate is closed). To avoid signal distortion, transitions between the open/closed states are smoothed using exponential weighting. The calculated gain is applied to the pressure signal.

**Figure 4:**
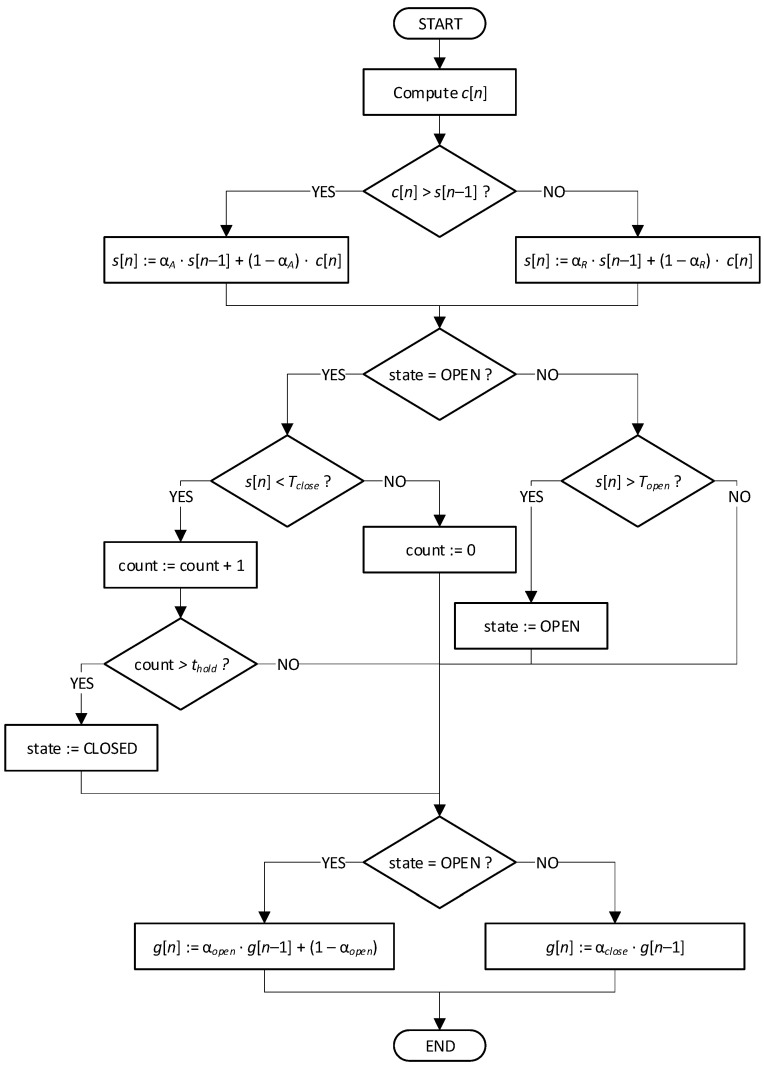
Flow chart of the algorithm for crosstalk detection and suppression.

The algorithm realizing crosstalk detection and suppression is explained with a flow chart in Figure 4. First, coverage values *c* are exponentially smoothed to obtain a detection metric *s*. Different smoothing factors are used depending on the values of *c* and *s*: α*_A_* (‘attack’) is smaller, to detect speech fragments as soon as possible; α*_R_* (‘release’) is larger, near unity, to introduce a degree of latency and to avoid frequent switching of the gate on/off. Next, the smoothed metric *s* is compared with the thresholds: *T_open_* and *T_close_*, depending on the current gate state (*T_open_* ≥ *T_close_*), and the state is switched if a condition is met. Additionally, a ‘hold’ period is introduced during the open-closed transition to avoid short-term state changes. Finally, the gain *g* is computed using exponential smoothing. The weighting factor α*_closed_* should be larger than α*_open_*, for the same reasons as described before.

**Figure 5:**
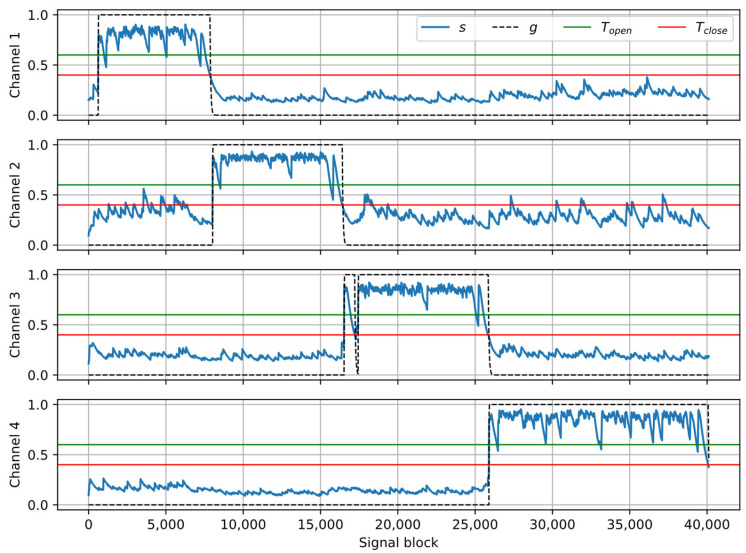
Example of signal analysis with the crosstalk gate algorithm: smoothed coverage *s*, threshold values *T_open_*, *T_close_*; output gain *g*.

Figure 5 shows the results of processing the example recording (the same as in Figure 2 and Figure 3) with the crosstalk suppression algorithm. The parameters used in the algorithm are tunable. The following values were used in the experiments: α*_A_* = 0.85, α*_R_* = 0.998, *T_open_* = 0.6, *T_close_* = 0.4, α*_open_* = 0.85, α*_closed_* = 0.98, *t_hold_* = 10 blocks. It can be observed that the smoothed coverage *s* is larger during the direct speech fragments than during crosstalk, hence the signal fragments related to the near speakers can be identified correctly and preserved, while crosstalk fragments are suppressed by setting the gain to zero.

**Figure 6:**
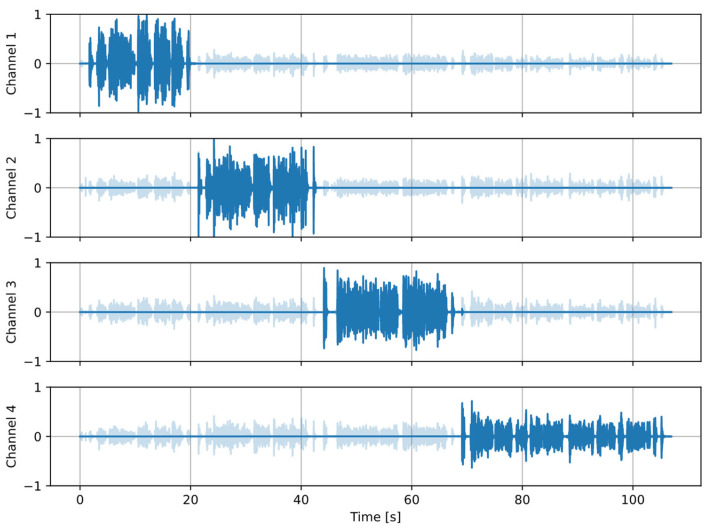
Example of crosstalk detection and suppression. Light color: the original signals, dark color: signals processed with the proposed algorithm.

Figure 6 shows the final processing results of the same test recording: the original signal with crosstalk, and the processed signal with crosstalk suppressed. It is evident that the proposed algorithm effectively removes crosstalk from the signal, without damaging direct speech.

## 3. Results

This section presents the recorded dataset, the results of validation of the proposed algorithm, and a comparison with a reference system. Additionally, the algorithm’s performance in an overlapping speech case is presented.

### 3.1. Evaluation Dataset

The proposed algorithm operates on signals recorded with AVSs. Each AVS is a four-microphone setup in a specific orientation. Publicly available datasets, commonly used for validation of similar algorithms, cannot be employed for this case, as they are recorded either with single-channel microphones or with microphone arrays in different setups. Therefore, a special dataset was created for the purpose of evaluating the presented algorithm.

A setup of four AVSs was used in the recordings. Each sensor was built using six identical, omnidirectional, digital MEMS microphones, positioned on the sides of a cube with a 10 mm side length. All sensors were calibrated in an anechoic room to compensate for the differences between the microphones [44]. Four of the six microphones were used in the algorithm; signals from the vertical axis were not used. All four sensors were connected to a personal computer with I^2^S-USB interfaces, and the computer synchronously recorded audio streams from all sensors, with a 48 kHz sampling rate and in a floating-point format.

**Figure 7:**
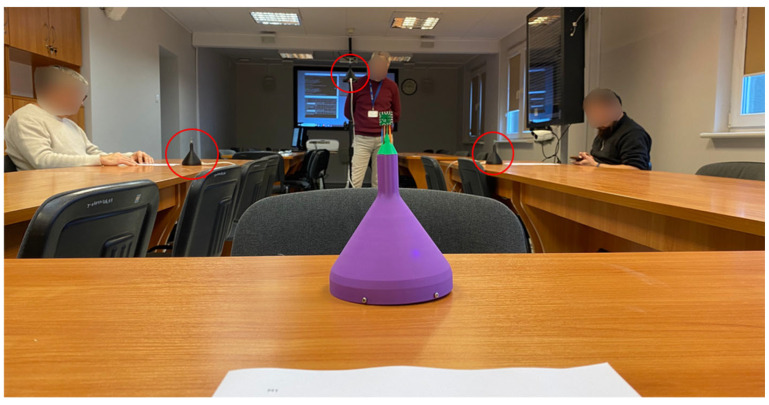
Recording of the dataset. Sensors: AVS1—in the front, AVS2—on the right table, AVS3—on the left table, AVS4—on the tripod in the back.

The recordings were performed in a typical conference room of size 14 m × 4.6 m × 2.5 m (cubature 161 m^3^). The reverberation time of the room was 0.7 s. Three sensors (AVS1, AVS2, AVS3) were placed on the tables, and the speakers were seated in front of them, the last sensor (AVS4) was fixed on a tripod, and the speaker was standing near it (Figure 7). Such a setup allowed for testing a scenario in which three seated speakers have mostly constant positions relative to the sensors, while the standing person moved slightly when speaking. Sensors AVS2-AVS4 were 1.4–2.0 m away from each other, while AVS1 was 3.4–3.7 m away from the other sensors.

Six male speakers participated in the recordings, four in each session. The speakers were given a written text, and each of them read the text in turns, AVS1 to AVS4. No overlapping speech segments were recorded. Fifteen recording sessions were performed, and a total of 44 min of material was collected. Each recording was then manually labelled to mark the start and the end of each segment in which a given near speaker was active.

### 3.2. Evaluation Metric

Each recording in the dataset was processed on a desktop computer, with the proposed algorithm implemented as Python 3.13 scripts, using the same algorithm parameters for all recordings. The processed audio streams were saved to files with a 16 kHz sampling rate, in 16-bit integer format.

The state-of-the-art metric used for the evaluation of algorithms that separate the desired signal (the target) from distortion (in this case: near speech from crosstalk) is the Scale-Invariant Signal-to-Distortion Ratio (SI-SDR) [45]. Calculation of this metric requires that the target signal be available:

(11) $$\text{SI-SDR}=10\log_{10}\left(\frac{{\left\|\frac{{\hat{s}}^{T}s}{{\left\|s\right\|}^{2}}s\right\|}^{2}}{{\left\|\frac{{\hat{s}}^{T}s}{{\left\|s\right\|}^{2}}s-\hat{s}\right\|}^{2}}\right)$$

where *s* is the target signal (clean speech), *ŝ* is the target estimate (the processed signal).

For the purpose of the experiments, the target signal was approximated by replacing all the recorded signal segments not containing near speech with noise recorded by the same sensor in the same acoustic conditions, when no speaker was active. The metric was calculated separately for each channel in every recording, so 60 cases were evaluated in total. The average SI-SDR values calculated for the signals obtained with the proposed algorithm were compared with the values obtained for the unprocessed recordings (the baseline), and a difference between these two results, ΔSI-SDR, was calculated. A higher value of ΔSI-SDR means that the algorithm is more effective in crosstalk removal.

### 3.3. Reference System

Evaluation of the proposed algorithm involves comparing the obtained results with a state-of-the-art reference system. As of late 2025, there is no known publicly available solution that would realize the complete pipeline for suppressing crosstalk in a multi-channel system, as described in this paper. However, such a system may be approximated with a speaker diarization engine, supplemented with the necessary signal processing blocks. The current state-of-the-art speaker diarization system is pyannote [46]. This AI-based model performs speaker diarization in single-channel recordings, without overlapping speech. Pyannote is usually the first choice in modern applications requiring speaker diarization. It is used, e.g., in the WhisperX system [47], which is de facto the current state-of-the-art ASR solution. However, pyannote does not operate on multi-channel signals, and it is not able to separate direct speech from crosstalk. Therefore, these operations must be implemented separately.

The processing pipeline in the reference system was realized as follows. For each recording, every channel was processed separately with pyannote (speaker diarization model 3.1), which produced a list of time indices and speaker labels for each recognized segment. Each detected segment is then assigned to a channel with the highest signal energy within the analyzed segment. This approach is an oversimplification, made solely for the algorithm evaluation. It works in the test dataset because all the sensors have identical sensitivity, and the level gain for each sensor during the recordings was the same. If such a system had to be implemented in a real system, a more robust selection of the near speaker channel would have to be developed. The result of the processing was a list of time indices that define the start and the end of each signal section containing direct speech. Next, each pressure signal was processed with the same crosstalk removal method as in the evaluated algorithm and with identical parameters, but using the detected time indices for opening and closing the gate instead of calculating the metric *s*. This way, output audio streams that may be compared with those produced by the evaluated algorithm were created. The SI-SDR metric was computed the same way as for the proposed algorithm.

### 3.4. Evaluation Results

The proposed algorithm was tested in four variants. Two factors were different between these versions. First, the speaker detection stage was performed either with a fixed or an automatic width of the azimuth range. In the initial version of the algorithm, only the maximum of each histogram mode was found, and the width of the azimuth range used for crosstalk detection and source separation was fixed at ±30° around the maximum (an optimal value found experimentally). In this paper, a method of dynamic selection of the azimuth range was introduced. Both versions of the speaker detection algorithm were tested and compared. Second, as described earlier in this paper, the source separation stage is not needed for crosstalk removal in speech signals without overlapping segments and without impulsive noise sources, as is the case in the recorded dataset. Therefore, the performance of the algorithm with and without the source separation stage was evaluated. As a result, four versions of the algorithm were tested on the dataset, and SI-SDR values were calculated and averaged over 60 test cases. Also, ΔSI-SDR was calculated relative to the scores of the unprocessed signals, and the mean values of differences were obtained. A similar approach was applied to the reference system.

**Figure 8:**
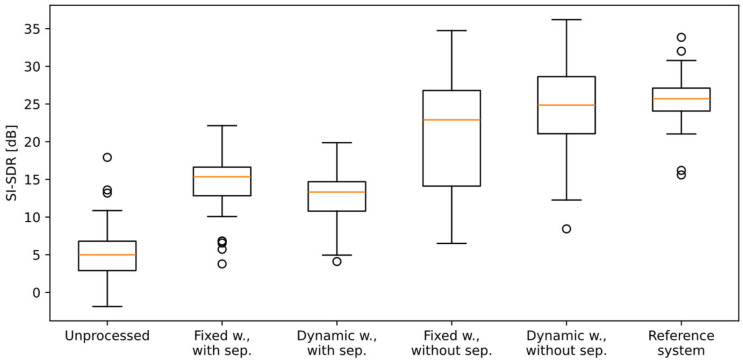
Box plot of the SI-SDR values obtained for various test cases. Orange line: the median (Q2); box: the quartiles Q1 to Q3; whiskers: Q1-1.5IQR to Q3+1.5IQR (IQR: inter-quartile range, Q3-Q1); circles: the fliers.

The results of the evaluation are presented in Table 1 and in Figure 8, and they will be discussed in the next Section of the paper. Additional analysis was performed for the individual AVSs, but no correlation was found between the position of the sensor and the obtained results.

Examples of the results obtained at the consecutive stages of the processing pipeline for one of the recordings from the dataset were presented as plots in Figure 2, Figure 3, Figure 5 and Figure 6. In the presented case, the mean ΔSI-SDR was 7.28 dB for the algorithm with source separation and 20.81 dB for the algorithm without the separation stage. For the reference system, the average ΔSI-SDR was 19.37 dB.

### 3.5. Algorithm Performance in the Presence of Overlapping Speech

**Figure 9:**
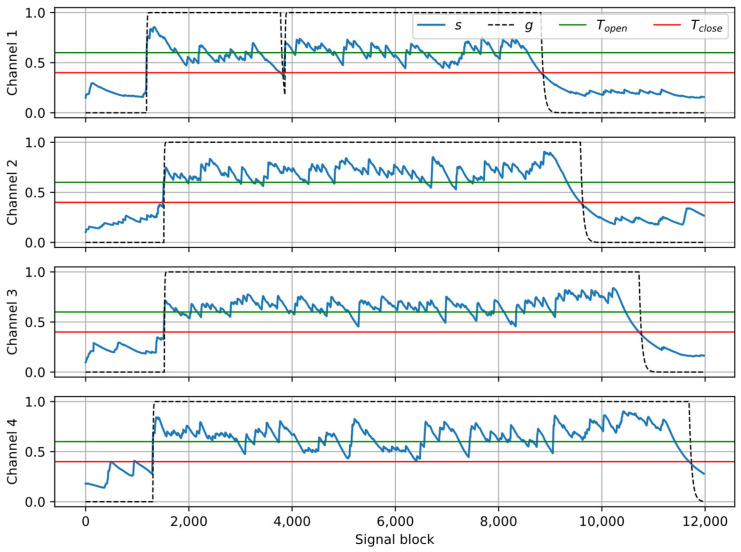
Example of crosstalk detection in the case of overlapping speech: smoothed coverage *s*, threshold values *T_open_*, *T_close_*; output gain *g*.

The formal evaluation of the proposed algorithm for crosstalk suppression, presented in the previous Subsection, was performed using recordings that did not contain overlapping speech, for reasons that are explained in the Discussion section. However, it is important to test the algorithm’s performance in the presence of overlapping speech to ensure that the algorithm only suppresses crosstalk and not direct speech. For this purpose, an additional, informal experiment was performed, using a recording in which four speakers were reading their texts concurrently. The length of each text was chosen so that the speakers finished reading at different times, starting with channel 1 and finishing with channel 4. Each channel is processed by the source separation algorithm, which separates near speech from crosstalk—this stage is necessary in the described scenario. The resulting signals are then processed with the gate algorithm, as before. The results of crosstalk suppression in this experiment are presented in Figure 9. Computing the SI-SDR metric was not possible in this case because a clear target signal is not available. Nevertheless, it can be observed that near speech segments were detected correctly in each channel, and crosstalk was also detected correctly after each speaker finished reading.

## 4. Discussion

The proposed algorithm for crosstalk detection and suppression in a multi-channel, multi-speaker system was evaluated, and the results were compared with the reference system built on pyannote, the state-of-the-art diarization system. The results of processing the recordings from the custom dataset, created especially for the algorithm evaluation, using the reference system, indicate that the diarization-based approach correctly identified each speaker and that the additional procedure for selecting the near speaker and removing crosstalk resulted in an SI-SDR increase by 20.49 dB, averaged from 60 test cases. This value serves as the baseline for further comparison.

Out of four versions of the evaluated algorithm, the one using a dynamic width of the azimuth range and omitting the source separation stage achieved the highest SI-SDR increase, by 19.53 dB. This result almost matches the one from the reference system, and there is no statistically significant difference between these two sets of results, as confirmed with the Wilcoxon statistical test (*p* = 0.44). The version with the azimuth range fixed to ±30° performed slightly worse (ΔSI-SDR = 16.2 dB). This confirms that the method of dynamic adaptation of the azimuth range, used in crosstalk detection, performs better than the fixed range version. It allows for more accurate separation of near speech from crosstalk, especially when the speaker’s position changes, which may lead to less accurate results with the fixed-width version. Also, the Wilcoxon test confirmed that the reference system and both algorithm versions mentioned above provide a statistically significant SI-SDR increase over the unprocessed signals (*p* < 0.05).

Two other versions of the algorithm performed the additional stage of source separation. As mentioned before, this stage is not necessary for the recordings from the created dataset, but it is essential when the processed signals contain overlapping speech and/or high-level background noise. The example of processing a recording containing overlapping speech, illustrated in Section 3.5, shows that thanks to the source separation algorithm, successful crosstalk detection and suppression are also possible in such cases. This is something that the reference system was not capable of.

Looking at the SI-SDR values obtained with the algorithm that includes the source separation stage, it can be observed that a large increase in this metric, compared with the unprocessed signals, was obtained (ΔSI-SDR = 7.54 dB for the dynamic width and 9.38 dB for the fixed width version). These differences are statistically significant (Wilcoxon test: *p* < 0.05). However, it is evident that these values are much lower than the ones obtained from the algorithm not performing source separation, and from the reference system. This is an effect of the SI-SDR calculation method. The recordings were made with omnidirectional sensors in a reverberant room. Therefore, the target signals used for SI-SDR calculation contain not only direct speech, but also sound waves reflected in a room (reverberation). The SI-SDR algorithm expects these reflected signal components to be present in the algorithm output. However, the source separation algorithm removes all signal components that originate from outside of the azimuth range of the near speaker. This includes not only crosstalk and noise, but also most of the reflected waves from the near speaker. The SI-SDR algorithm treats this situation as an impairment of the target signal. Hence, the calculated scores are lower, although direct speech is preserved in the output signal. This also explains why the dynamic width version of the algorithm received a lower score than the fixed width version: more reflected sound waves are removed in the dynamic width version, leading to a lower SI-SDR score.

Despite the issues discussed above, it was decided to use SI-SDR for the algorithm evaluation, as it is the metric widely used by most of the researchers, and there is no other standard metric that would avoid the discussed problems. It would be possible to perform the recordings in an anechoic room, and it is expected that the SI-SDR for the algorithm performing the source separation would be higher. However, the purpose of the experiments was to evaluate the algorithm in real-world conditions, in a reverberant space. The conclusion is that the SI-SDR metric does not provide full information on the suitability of the processed signals for the speech recognition task. The experiments presented in the previous publication [41] indicate that the proposed source separation procedure does not impair speech recognition accuracy, and these observations may be extrapolated to the algorithm described here. Listening to the recordings processed with the algorithm confirmed that direct speech remains intelligible, and the preliminary results of ASR testing with WhisperX 3.3.1 confirmed that the proposed algorithm allows for accurate speech recognition. Formal tests on this subject are planned for future research, because testing the ASR accuracy is outside of the scope of this paper. It may be concluded that the proposed algorithm in the version performing source separation provides a statistically significant increase in SI-SDR over the unprocessed signals.

The validation tests were performed using recordings that contained no overlapping speech segments. Informal tests of processing the recordings containing overlapping speech fragments were also performed, and the example presented in this paper confirms that the source separation stage allows for correct crosstalk detection in this case. Formal evaluation of the algorithm performance in the presence of overlapping speech is a complicated problem. In fact, such an experiment would test two concepts at a time: source separation and crosstalk detection. The source separation algorithm was evaluated in the previous publication [41], and it was shown that it works as expected. This paper focused on the evaluation of the crosstalk detection and suppression algorithm only. Moreover, the SI-SDR metric requires a clean target signal, which cannot be obtained from the recordings if overlapped speech is present. Algorithms in this scenario are usually validated using synthetic test signals, and in this paper, the intent was to evaluate the algorithm’s performance using real-world recordings. Also, the reference system used for evaluation in this scenario would have to be extended with a state-of-the-art source separation model. Because of all these issues, in this paper, it was decided to focus on the case with one speaker active at a time, and to leave the overlapping speech scenario for future publications.

The algorithm version using the dynamic azimuth range and not using the source separation stage obtained a comparable SI-SDR score to the reference system, built on pyannote. Therefore, a question may be asked: why use the proposed algorithm instead of employing the reference system directly for crosstalk suppression in a multi-sensor system? The main advantages of the reference system are that it operates on single-channel signals that may be recorded with simple microphones and that it provides an all-in-one solution for crosstalk suppression, diarization, and ASR. However, there are several practical advantages of using the proposed algorithm in the presented scenario. First, the presented algorithm operates on signals both without and with overlapping speech, while the reference system needs a separate model for speaker separation. Second, the proposed algorithm has tunable parameters that allow adjusting its performance to the acoustic conditions, while the reference system is a black box approach. Third, the proposed method allows for relocating the crosstalk suppression procedure to the preprocessing stage, which may be performed locally, on the same machine that performs the recordings, offloading this task from the ASR system, usually working ‘in the cloud’. This may lead to a reduction in the processing time and in costs. Fourth, the proposed algorithm is based on DoA analysis, hence it is robust to variations in the speech signal. The reference solution is based on speech features extracted by the model, which leads to the risk of incorrect diarization if two speakers have similar voices or if the tone of the speaker’s voice changes. This effect was not observed in the test dataset, but it was detected earlier in several cases of processing spontaneous speech recordings with WhisperX. Fifth, the proposed method identifies near speech and crosstalk, while the reference system only performs diarization; the decision whether the detected signal segment is direct speech or crosstalk requires an additional procedure, more refined than the simplified one used only for the algorithm evaluation in this paper. To conclude, the algorithm proposed here may be a viable solution for practical applications of multi-sensor, multi-speaker systems involving ASR.

In the experiments presented here, a separate sensor was used for each speaker. However, the speaker detection algorithm is not limited to a single speaker. In practice, up to four speakers may be detected using a single AVS, provided that all speakers are sufficiently close to the sensor (<2 m) and that there is a sufficient spatial separation between the speakers (at least 45° is recommended). If the distance between a speaker and a sensor is above 2 m, a separate AVS should be used for that speaker. This way, the system configuration may be adapted to the speaker layout in a room, allowing for a reduction in the number of sensors and the processing time. The maximum number of sensors is only limited by the hardware used, not by the algorithm. During the experiments, it was found that the distances between speakers did not directly affect the algorithm’s performance. The main factor here is the crosstalk level, which depends on many factors, such as the loudness of speech and the amount of sound wave reflections at various points in a room. The experiments presented in this paper were performed in a typical conference room to evaluate the algorithm in acoustic conditions similar to a small courtroom. In this scenario, sound reflections had a larger influence on the crosstalk level than the distance between the sensors.

The testing scenario involved three speakers seated at a table, and one speaker standing and moving within a small range. A scenario in which a speaker moves freely across the room may also be considered. The algorithm may be modified by performing the analysis on shorter fragments of the recording (e.g., 10 s), and assuming that the speaker’s position is constant within the segment, but it may change between the segments. Additionally, the algorithm would have to be supplemented with a source tracking method, and it must also assume that the speaker can move between the channels. Such a scenario will be investigated in future research.

## 5. Conclusions

The proposed algorithm solves the problem of crosstalk in a multi-sensor system in a reverberant room, when multiple speakers are active. The solution is based on the analysis of signals recorded with AVSs. A pipeline of signal processing blocks: detection of speakers’ positions, source separation, crosstalk detection, and crosstalk suppression, was described. The experiments were performed using the dataset created especially for this purpose. It was shown that the algorithm successfully suppresses crosstalk in multi-channel audio recordings. An increase in SI-SDR was obtained: 7.54 dB for the algorithm version performing source separation and 19.53 dB for the version without the separation stage. The latter result is close to the value obtained from the reference system, built on the pyannote diarization model (20.49 dB). Unlike the reference system, the proposed algorithm with the source separation stage employed is able to suppress crosstalk also in the presence of overlapping speech. The proposed algorithm is based on DOA analysis and, contrary to the methods based on the extraction of speech features, it does not depend on speaker-dependent factors such as gender, age, manner of speaking and language. The main condition for proper operation of the algorithm is a sufficient SNR of the near speech.

The proposed method is intended for practical ASR applications in reverberant spaces, such as small courtrooms or conference rooms, with multiple speakers positioned across the room. A practical system may work as follows. Several AVSs are placed in a room, depending on the number of speakers and their positions. The algorithm analyzes the recorded audio streams on a local computer; it detects the number of active speakers and directions of the near speakers relative to the sensors. Next, signal fragments representing direct speech are detected, and crosstalk is suppressed. The resulting audio streams without crosstalk are then sent to an ASR system operating ‘in the cloud’, the system performs speech recognition for each signal channel, then the results are merged into a complete transcript of the recording. Such a system may be useful in practical scenarios that require an accurate report of the discussion, but multiple sensors are needed to achieve a good SNR, and crosstalk must be removed from the final transcript.

## Figures and Tables

**Table 1:** SI-SDR values (in dB) and their difference relative to the unprocessed signals, mean ± standard deviation, for various test cases.

Test Case	SI-SDR	ΔSI-SDR
Unprocessed signals	5.13 ± 3.52	–
Fixed width, with separation	14.51 ± 3.50	9.38 ± 4.16
Dynamic width, with separation	12.67 ± 2.98	7.54 ± 4.06
Fixed width, without separation	21.33 ± 7.09	16.20 ± 7.40
Dynamic width, without separation	24.66 ± 5.29	19.53 ± 5.15
Reference system	25.62 ± 3.04	20.49 ± 3.17

## Data Availability

The data presented in this study are openly available in repository at the URL: https://multimed.org/datasets/crosstalk/ (accessed on 17 October 2025).

## Abbreviations

The following abbreviations are used in this manuscript:

AI	Artificial Intelligence
ASR	Automatic Speech Recognition
AVS	Acoustic Vector Sensor
DNN	Deep Neural Networks
DOA	Direction of Arrival
SI-SDR	Scale Invariant Source-to-Distortion Ratio
SNR	Signal-to-Noise Ratio
